# Favipiravir and Its Structural Analogs: Antiviral Activity and Synthesis Methods

**DOI:** 10.32607/actanaturae.11652

**Published:** 2022

**Authors:** I. D. Konstantinova, V. L.Andronova, I. V. Fateev, R. S. Esipov

**Affiliations:** Shemyakin and Ovchinnikov Institute of Bioorganic Chemistry, Russian Academy of Sciences, Moscow, 117997 Russia; FSBI «National Research Centre for Epidemiology and Microbiology named after the honorary academician N.F. Gamaleya» of the Ministry of Health of Russia, Moscow, 123098 Russia

**Keywords:** 6-fluoro-3-oxopyrazine-2-carboxamide, favipiravir, pyrazine-2-carboxamide, influenza, SARS-CoV-2

## Abstract

1,4-Pyrazine-3-carboxamide-based antiviral compounds have been under intensive study for the last 20 years. One of these compounds, favipiravir (6-fluoro-3-hydroxypyrazine-2-carboxamide, T-705), is approved for use against the influenza infection in a number of countries. Now, favipiravir is being actively used against COVID-19. This review describes the *in vivo *metabolism of favipiravir, the mechanism of its antiviral activity, clinical findings, toxic properties, and the chemical synthesis routes for its production. We provide data on the synthesis and antiviral activity of structural analogs of favipiravir, including nucleosides and nucleotides based on them.

## INTRODUCTION

Infectious diseases caused by both new, previously unknown viruses and re-emerging, known viruses, including their new variants, are one of the main causes of high mortality, mass epidemics, and pandemics. Three or four previously unknown viruses which are dangerous to humans are discovered annually [1]. The free movement of people increases the risk of a rapid spread of a viral infection among the population. In addition, viruses dangerous to humans can be transmitted by the insects or rodents that accompany our various goods. Furthermore, the ever-increasing interaction between humans and nature periodically leads to the emergence of diseases that are caused by zoonotic viruses capable of infecting humans: i.e., of overcoming the species barrier, or new variants of zoonotic viruses that have acquired the ability to infect humans through genetic variability. These viruses include the human immunodeficiency virus, influenza virus (H1N1), the highly pathogenic avian influenza virus (H5N1), Hendra virus (HeV), Zika, dengue, and yellow fever viruses, the Ebola virus (EBOV), severe acute respiratory syndrome coronavirus (SARSCoV- 1) [2], and COVID-19 (SARS-CoV-2) [3]. Viruses that can not only infect but also effectively be transmitted from person to person can cause serious outbreaks and/or epidemics/pandemics [1].

Obviously, the development of safe and highly selective broad-spectrum antiviral agents is a must if we want to combat new, resistant forms of known viral infections. Of particular interest are synthetic analogs of natural nucleosides and nucleotides, because they have been used for a long time to diagnose and treat various infectious diseases and have also retain considerable biological and pharmaceutical potency [4, 5].

**Fig. 1:**
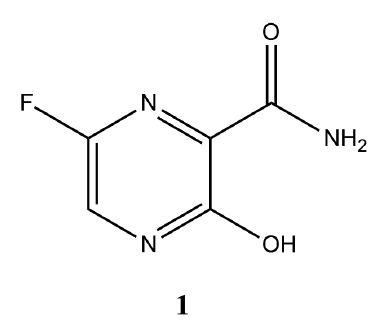
Chemical structure of favipiravir (T-705, 6-fluoro-3-hydroxypyrazine-2-carboxamide)

Favipiravir (6-fluoro-3-hydroxypyrazine-2-carboxamide, or T-705) (**1**) (*Fig. 1*) is a synthetic analog of 1,4-pyrazine-3-carboxamide. Its activity against the influenza virus A/PR/8/34 (H1N1) was discovered in the research laboratory of Toyama Chemical Co., Ltd [6].

Later, favipiravir was found to exhibit selective activity against a wide range of unrelated RNA viruses, including socially significant and especially dangerous pathogens, such as orthomyxoviruses (the influenza viruses A, B, and C), flaviviruses (yellow fever, West Nile, and Zika viruses), togaviruses (the Eastern, Western, and Venezuelan equine encephalitis viruses, the Chikungunya virus), arenaviruses (the Lassa fever and Junin viruses), filoviruses (the Ebola virus), paramyxoviruses (the respiratory syncytial virus and human metapneumovirus), rhabdoviruses (the rabies virus), etc., but not to be active against DNA viruses [7, 8, 9, 10].

## ANTI-INFLUENZA ACTIVITY OF FAVIPIRAVIR

Favipiravir is an effective inhibitor of the reproduction of the human influenza viruses A, B, and C and it exhibits activity against strains resistant to all anti-influenza drugs of practical importance: neuraminidase inhibitors (oseltamivir, zanamivir, laninamivir, peramivir); M2 protein inhibitors (amantadine and rimantadine with a 50% effective concentration (EC_50_) in a range of 0.014 to 0.55 μg/mL [11 , 12, 13] and against the pig viruses A/H2N2 and A/H4N2, the highly pathogenic avian virus A/H5N1, and the new virus A/H7N9. The toxic effect of favipiravir on a MDCK cell culture have proved insignificant, and the CC50 (50% cytotoxic concentration) was not achieved even at a concentration of 2,000 µg/mL, which is an indication of the compound’s ability to highly selectively inhibit the replication of influenza viruses [12 , 13, 14, 15].

**Table:** The *in vivo *antiviral activity of favipiravir administered orally against some influenza virus strains

Influenza virus strain	Activity
A/Victoria/3/75 (H3N2)	duces a 70 and 100% survival rate in mice, respectively (100% lethality of mice in the control group). The pulmonary viral load in mice one day after the onset of treatment (100 mg/kg/day, 4 times a day) is reduced by more than 1 lg TCID50/g. In the group treated with oseltamivir (20 mg/kg/day, 2 times a day for 5 days), the survival rate was 50% and the reduction in the pulmonary viral titer was 0.1–0.2 lg [11]
A/Duck/MN/1525/81 (H5N1)	Upon 100% lethality in the control group, administration of favipiravir (30 mg/kg/day, 4 times a day for 5 days) provides 100% survival rate of mice, and oseltamivir (20 mg/kg/day, 2 times a day for 5 days) provides a 20% survival rate in mice. The 100% protective effect of favipiravir at a dose of 300 mg/kg/day is fully preserved at a delay of 36 h in the onset of treatment and decreases to 90% at a delay of 48–72 h [11]
A/PR/8/34 (H1N1)	Increased survival rate of mice from 21.4 to 87.5% compared with that in the control, untreated group, a reduction in the pulmonary viral titer by 3 lg PFU/lung (100 mg/kg/day, 4 times a day within 5 days), and prevention of death of mice were achieved as the single dose (200 mg/kg/day) was increased; in 80% of mice, the pulmonary viral titer was below the detection threshold [6]
A/Vietnam/UT3040/04 (VN3040) (H5N1) highly pathogenic for mice	Mortality in the control group was 100%. Administration of favipiravir (300 mg/kg/day, 2 times a day) provided a 50 and 100% survival rate at a 5- and 8-day course, respectively. At an 8-day course, the infection was asymptomatic and the efficacy in animal protection was preserved even at a delay of 72 h in the first drug administration. As the dose of favipiravir was reduced to 100 mg/kg/day (8-day course), the survival rate of the animals decreased to 90%; and at a delay of 48 and 72 h in the onset of treatment, the survival rate of the animals decreased to 60 and 25%, respectively. Administration of favipiravir stops tracheitis and bronchitis, dose-dependently decreases the production of pro-inflammatory cytokines and the affected lung area, and significantly reduces the infectious titer of the virus in the lungs and brain [14]
VN1203-H274Y is a oseltamivir- resistant variant of the A/Vietnam/UT3040/04 (VN3040) virus that is highly pathogenic for mice	Administration of favipiravir to mice (100 and 300 mg/kg/day, 2 times a day) for 8 days provided a 50 and 100% survival rate of animals, respectively, with 100% lethality of the animals in the control group [14]

The high activity of favipiravir *in vivo *was confirmed in a model of lethal influenza infection in mice that had received the drug orally (*Table*). Administration of favipiravir to animals infected with type A influenza virus was shown to provide for a dose-dependent decrease in the pulmonary viral titer and animal mortality. The therapeutic efficacy of favipiravir varies depending on the influenza virus subtype and strain.

Importantly, the protective effect of favipiravir does not depend on the virus sensitivity to oseltamivir [14]. The potentiating effect of the interaction between favipiravir and oseltamivir has been shown in mice infected with the A/H1N, A/H3N2, and A/H5N1 virus subtypes [16, 17]. In addition, the combination of favipiravir and oseltamivir is also effective against infections caused by the highly-resistant-to-oseltamivir influenza virus strain A/Mississippi/03/2001 (H1N1) H274Y. In this case, oseltamivir was not effective even when it was used at a dose of 100 mg/kg/day (administered twice daily for 5 days). Upon simultaneous administration of oseltamivir (50 mg/kg/day) and favipiravir (12.5 mg/kg/day) at doses that were not protective when given alone (100% mortality), all the animals survived [18].

The synergistic effect of favipiravir and another inhibitor of influenza virus neuraminidase, piramivir, was also demonstrated in experiments in mice infected with the pandemic influenza virus A/California/04/2009 (H1N1) [19].

## MECHANISM OF THE ANTIVIRAL ACTIVITY OF FAVIPIRAVIR

**Fig. 2:**
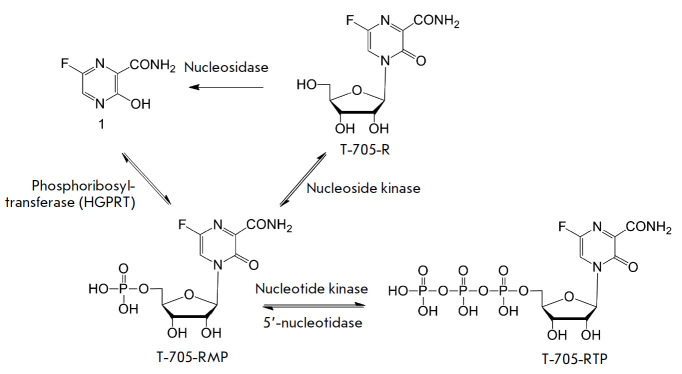
Formation of the active form of favipiravir

The mechanism of favipiravir action has been exhaustively studied in the influenza virus. Favipiravir has been shown to act on the RNA-dependent RNA polymerase (RdRp) of the influenza A virus, which comprises the virus-encoded proteins PB1, PB2, and PA. A metabolite of favipiravir, favipiravir-4-ribofuranosyl- 5’-triphosphate (T-705-RTP), exhibits biological activity. The intracellular transformation of favipiravir resulting in the active metabolite involves only cellular enzymes. Favipiravir is first converted by hypoxanthinguanine phosphoribosyl transferase (HGPRT) to ribose 5’-monophosphate (T-705-RMP) and then metabolized to the triphosphate form by cellular kinases [20, 21]. T-705-RTP is recognized by viral RdRp, effectively competing with the natural substrates GTP and, to a lesser extent, ATP, and is included in the growing RNA chain [11, 14, 22]; it also inhibits RdRp activity, which leads to the total suppression of virus- specific RNA synthesis (transcription and replication of the viral genome). A scheme of metabolic transformations of favipiravir is shown in *Fig. 2* [20]. It is important to emphasize that favipiravir does not significantly affect DNA and cellular RNA synthesis, which is explained by a lack of the suppressive effect of T-705 on cellular DNA polymerases (α, β, and γ) and DNA-dependent RNA polymerase II [11].

Until recently, there had been only two reports of a slight decrease in the antiviral effect of T-705 against the influenza viruses A/H3N2 and A/H5N1 (1.8-fold and 1.5-fold) with the V43I mutation in the PB1 polymerase subunit (one of the proteins that form the ribonucleoprotein) [23, 24].

D. Goldhill et al. generated an A/H1N1 influenza strain highly resistant to favipiravir (the virus sensitivity decreased 30-fold) [25]. The decrease in the sensitivity was caused by a combination of two mutations in RdRp: K229R in the PB1 subunit and P653L in the PA subunit. The K229R mutation causes resistance to favipiravir, but critically (by a factor of 30) it reduces the activity of RdRp and the efficiency of virus reproduction. The P653L mutation in the PA subunit is compensatory and restores the polymerase activity associated with PB1 without reducing resistance, as well as normalizes the kinetics of mutant virus replication. The role of the combination of K229R + P653L mutations in the development of resistance to favipiravir was confirmed for two more influenza A virus subtypes (H3N2 and H7N9). Interestingly, the introduction of the K229R substitution in PB1 or a combination of PB1/K229R+PA/P653L substitutions reduces the mutagenic effect of favipiravir; i.e., the fidelity of RdRp in virus replication increases: the produced RNA contains significantly fewer mutations even in the presence of T-705 at a high concentration of 100 μM compared to wild-type RdRp; incorporation of T-705 into the growing viral RNA during *in vitro* replication also decreases [25].

The antiviral effect of favipiravir against a large number of RNA viruses may be partially explained by its ability, after transformation into T-705-RTP, to integrate into the synthesized viral RNA and bind to conserved RdRp domains, thereby inhibiting virus replication. For example, the use of other viral models with an RNA-positive genome enabled the generation of virus strains resistant to favipiravir and identification of the molecular mechanism of drug resistance. The key mutation, K291R, in the Chikungunya virus (togavirus) was localized in nsP4 (RdRp) and, like the K229R mutation in the influenza virus, occurred in the highly conserved motif F of nsP4, which possesses RNA polymerase activity [26]. A similar genetically engineered K159R mutation in motif F of 3D (RdRp) in the Coxsackie B3 virus (picornavirus) fatally reduced the activity of the purified viral RdRp and was lethal. As in the influenza virus, a compensatory A239G mutation in RdRp was required to restore the viability of the mutant virus [27].

On the other hand, a number of researchers believe that at least two consecutive inclusions of T-705-RMP are required to stop RNA elongation. Therefore, the central mechanism of virus replication inhibition may be termination of RNA synthesis at high favipiravir concentrations and the ability to induce lethal mutagenesis at low favipiravir concentrations [28]. This has been shown in experiments with the influenza A(H1N1) [17, 29], hepatitis C [30], West Nile [31], dengue [32], and Ebola [33] viruses.

The mechanism of lethal mutagenesis is explained by the concept of error threshold: if the mutation rate during genome replication is above the error threshold, this is equivalent to a loss of hereditary information [34]. Most RNA-containing viruses are characterized by a high mutation rate, due to the lack of a mechanism that corrects errors during viral genome replication [35]. Hypermutability promotes rapid adaptation of viruses to certain adverse environmental changes; e.g., it allows rapid development of resistance to antiviral drugs. However, in the presence of a hypermutator during viral genome replication, the mutation rate exceeds the threshold level and defective genomes are synthesized, which leads to the formation of non-viable viral particles. Phenotypically, this is expressed as a significant decrease in the infectivity of a new virus generation (ratio of the titer of infectious viral particles to the number of viral genome copies) [36].

*In vitro *experiments have revealed that a decrease in the number of infectious particles of the influenza A/H1N1 virus in the presence of favipiravir does not correlate with a decrease in the number of RNA copies (viral genomes), which is an indication of preserved activity of the transcription complex and an increase in the content (%) of defective viral particles in the population. Analysis of the *NP *gene sequence showed a dose-dependent increase in the rate of mutations, mainly transitions (G→A and C→U), and a shift in the nucleotide profiles of individual clones [29]. Hypervariability of the influenza A/H5N1 virus was also observed in experiments *in vivo *during an infection of mice treated with favipiravir compared with the control group and mice treated with oseltamivir [17].

## EFFICACY OF FAVIPIRAVIR AGAINST THE SARS-CoV-2 CORONAVIRUS INFECTION

In 2014, favipiravir (under the brand name Avigan®) was approved in Japan for the treatment of new or re-emerging pandemic influenza virus infections, although its use was limited to cases where licensed influenza drugs were ineffective or insufficiently effective (http://www.toyama-chemical.co.jp/eng/news/ news140324e.html) [37].

Since the outbreak of the novel coronavirus SARSCoV- 2 epidemic in China at the end of December 2019 and its rapid spread around the world, dozens of known pharmaceuticals with antiviral activity [38 , 39, 40], including favipiravir [3, 41], have been tested as possible therapeutic agents for the treatment of patients infected with COVID-19.

*In vitro*, SARS-CoV-2 was significantly less sensitive to favipiravir than the influenza virus is. Favipiravir activity against the clinical isolate nCoV- 2019BetaCoV/Wuhan/WIV04/2019 manifested itself when used at a concentration of 61.88 µM (EC_50_), and the maximum studied concentration of 400 µM was non-toxic to a Vero E6 cell culture (CC50 > 400 µM, selectivity index SI > 6.46) [42]. In another study, favipiravir proved ineffective against the SARS-CoV-2 clinical isolate BetaCoV/HongKong/VM20001061/2020 even at a concentration of 100 µM [43].

It is important to note that investigation of the effect of favipiravir on animals at doses equivalent to the proposed human treatment regimens revealed its embryotoxicity: in rats, there was fetal death in the early stages of embryogenesis, a decrease in the live fetal body weight and the number of live fetuses, decreased litter survival 4 days after birth, and reduced weight gain. In addition, favipiravir was found to be teratogenic in mice, rats, rabbits, and monkeys [3]. Given the high risk of teratogenicity and embryotoxicity of favipiravir, no human clinical trials have involved pregnant or lactating females and trial participants have been required to abstain from unprotected sex during trials and for 90 days after the last dose of the drug. Therefore, the risks to humans remain unknown and the use of favipiravir remains under strict supervision, which limits its use, especially in pregnant females [10].

The ribose-5’-triphosphate metabolite of favipiravir is known to be a substrate for human mitochondrial RNA polymerase [44]. *In vitro *incorporation of T-705-RTP into mitochondrial RNA was shown to have no toxic effect on human mitochondria; i.e., it did not lead to chain termination or inhibition of DNA-dependent RNA polymerase activity. However, favipiravir should be used with caution because it may exert an indirect toxic effect on mitochondria [44].

Forty-seven clinical trials (of which 17 are completed) on the efficacy of favipiravir for the treatment of COVID-19 had been registered on the clinicaltrials. gov site as of November 27, 2020. Trial protocols for the use of favipiravir in adult COVID-19 patients typically indicate the following dosage: a loading dose of 1,600 or 1,800 mg twice daily on day 1, then a maintenance dose of 1,200–2,000 mg daily in 2, 3, or 4 doses for the next 4–13 days. The results of several clinical trials of favipiravir for COVID-19 point at critical factors affecting the treatment outcome; in particular, loading doses < 45 mg/kg per day, older age, and baseline disease severity.

We will describe the results of several clinical trials conducted in China and the Russian Federation.

An open-label, randomized, multicenter study of 236 adults with moderate, severe, or critical COVID-19 pneumonia was conducted in China (ChiCTR2000030254): 116 patients received favipiravir (1,600 mg orally, twice daily on day 1, then 600 mg orally twice daily for 7–10 days), and 120 patients received umifenovir (Arbidol®; 200 mg 3 times a day for 7–10 days). The rate of clinical recovery at day 7 in patients with moderate COVID-19 pneumonia was 61% (71/116) in the favipiravir group versus 52% (62/120) in the umifenovir group; in patients with severe or critical COVID-19, this rate was 16% versus 0%, respectively. Relief for pyrexia and cough was achieved faster in the favipiravir group [45].

An open-label, controlled trial of the efficacy of favipiravir for the treatment of COVID-19 was conducted at the Third People’s Hospital of Shenzhen, China (Chinese Clinical Trials Registry, ID: ChiCTR2000029600), between January 30 and February 14, 2020 [46]. The trial included patients aged 16 to 75 years with a laboratory-confirmed diagnosis of coronavirus infection and clinical manifestations of the disease for no more than 7 days (N = 35). Patients who had initially received antiviral therapy with lopinavir/ritonavir before January 30, 2020, were included in the control group (N = 45). All baseline characteristics of the clinical status of the patients in the groups were comparable. Favipiravir was used orally: 1,600 mg twice daily on day 1, then 600 mg twice daily on days 2 to 14 + α-interferon (5×10^6^ IU twice daily as an aerosol inhalation). The patients in the control group received lopinavir/ritonavir (400 mg/100 mg twice daily for 14 days + α-interferon (5×10^6^ IU twice daily as aerosol inhalation). In the favipiravir group, the mean viral clearance time (4 days) was shorter than that in the control group (11 days) and there was also a significant improvement in chest CT compared with that in the control group, 91% vs 62%, respectively. In this trial, favipiravir demonstrated the best therapeutic effect, as measured by COVID-19 progression and viral clearance.

In the Russian Federation, in an interim pilot phase of an open-label, randomized, multicenter phase II/III clinical trial comparing the efficacy of Avifavir (favipiravir) and standard treatment (SOC) in 60 hospitalized adult patients (aged 60 years and older) with moderate COVID-19 pneumonia (Russia, NCT04434248) the following dosage regimens were used: favipiravir 1,600 mg orally twice daily on day 1, then 600 mg twice daily on days 2–14 (group 1, N = 20) or 1,800 mg twice daily on day 1, then 800 mg twice daily on days 2–14 (group 2, N = 20). In group 3 (SOC, control), 15 patients received hydroxychloroquine or chloroquine, one patient received lopinavir/ritonavir, and four patients received no etiotropic treatment [47]. The virological response to favipiravir in groups 1 and 2 was as follows: viral clearance was achieved in 25/40 (63%) patients on day 4 and in 37/40 (93%) patients by day 10. The same indicators in group 3 (SOC) were 6/20 (30%) and 16/20 (80%) patients, respectively. The mean time to body temperature normalization ( < 37°C) was 2 days in groups 1 and 2 and 4 days in the SOC group. By day 15, chest CT findings had improved in 90% (36/40) of patients treated with favipiravir versus 80% (16/20) of patients treated with SOC. Mild to moderate adverse drug reactions to favipiravir (diarrhea, nausea, vomiting, chest pain, and elevated hepatic transaminases) were reported in 7/40 (18%) patients and resulted in discontinuation of the study drug in 2/40 (5%) patients. Thus, the mean duration of favipiravir administration was 10.9 ± 2.8 days.

Between May 21 and August 10, 2020, an open, randomized, multicenter phase 3 study was conducted in the Russian Federation [48] to evaluate the efficacy and safety of favipiravir tablets (Areplivir, PROMOMED RUS LLC, Russia) compared to the Standard of Medical Care in patients hospitalized with moderate COVID-19 pneumonia (ClinicalTrials. gov ID: NCT04542694). The study was conducted in four medical institutions: State Clinical Hospital No. 50 (Moscow), Regional Clinical Hospital (Ryazan), City Hospital No. 40 of Kurortny District (Saint- Petersburg), and Smolensk Clinical Hospital No. 1 (Smolensk).

Two hundred patients aged 18 to 80 years with an established diagnosis of moderate SARS-CoV-2 infection were randomized in a 1:1 ratio. The patients in group 1 received favipiravir 1,600 mg twice daily (8 tablets at a time, a total of 16 tablets per day) on day 1 and then 600 mg (3 tablets) twice daily (6 tablets per day) on days 2–14. The patients in group 2 received standard therapy, but not favipiravir (hydroxychloroquine with or without azithromycin, chloroquine, lopinavir/ritonavir, or other recommended regimens). The rate of clinical status improvement by day 10 evaluated using the WHO categorical ordinal scale of clinical status improvement was 27% in group 1 and 15% in group 2. The virus clearance rate by day 10 – the percentage of patients with COVID-19 elimination according to PCR – was 98% (group 1) and 79% (group 2). The CT extent of lung damage (decrease in the lesion size) compared with the baseline level was 60% in group 1 and 40% in group 2. Mortality in both groups was 0%. During the treatment in both groups (200 patients), there was no need to transfer patients to an intensive care unit or use non-invasive ventilation or mechanical ventilation (MV).

Despite the side effects, the efficacy and wide range of antiviral activity of favipiravir make it a promising antiviral compound.

These results led to the approval of favipiravir for the treatment of the coronavirus infection (COVID-19) in several countries, including China [49] and India [50].

In the Russian Federation, favipiravir has been used since 2020 as an etiotropic drug for a mild to moderate coronavirus infection (COVID-19) [51, 52]; it is also included in the List of Vital and Essential Medicines for Medical Use for 2021 [53] (https:// mine-med.ru/archive/p2021p1.pdf). Favipiravir is produced in the form of film-coated tablets under the trade names Avifavir (Kromis), Areplivir (Promomed Rus), Favipiravir (Alium), Covidolek (Nanolek), Favibirin (Pharmasintez), and Coronavir (Technology of Medicines) [54]. In addition, in 2021, the first domestic drug for intravenous administration (Areplivir, Promomed Rus) received marketing authorization from the Ministry of Health of the Russian Federation [55].

Currently, structural analogs of favipiravir are under study for antiviral activity. This is especially important when many RNA virus diseases lack approved antiviral drugs or effective vaccines, and most interventions are limited to supportive care.

## STRUCTURAL ANALOGS OF FAVIPIRAVIR EXHIBITING ANTIVIRAL ACTIVITY

**Fig. 3:**
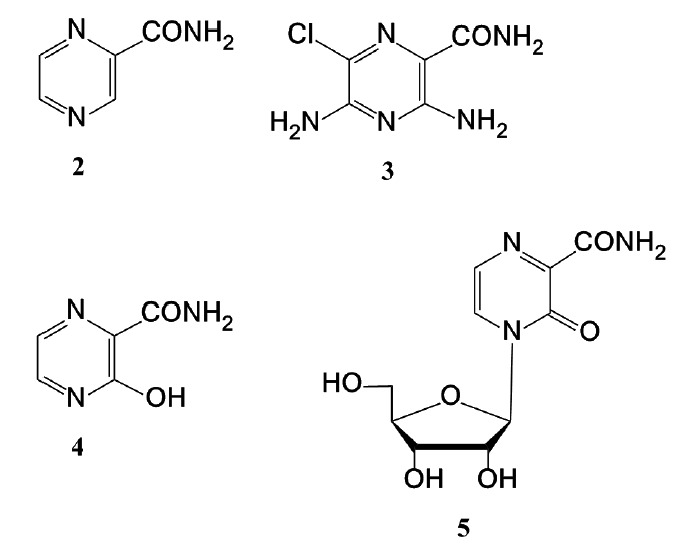
Some structural analogs of favipiravir: **2 **– 2-pyrazinecarboxamide; **3 **– 3,5-diamino-6-chloro-2-pyrazinecarboxamide;** 4 **– 3-hydroxypyrazine-2-carboxamide (T-1105); **5 **– 3-oxo-4-(β-*D*-ribofuranosyl)-2-pyrazinecarboxamide (T-1106)

Structural analogs of favipiravir include the following compounds (*Fig. 3*):

Of particular interest are T-1105 (**4**) and T-1106 (**5**), synthesized at the research laboratory of Toyama Chemical Co., Ltd. The antiviral activity of these analogs against influenza virus A/PR/8/34 (H1N1) was established in 2009 during *in vitro *screening of a chemical library of compounds [7].

**Fig. 4:**
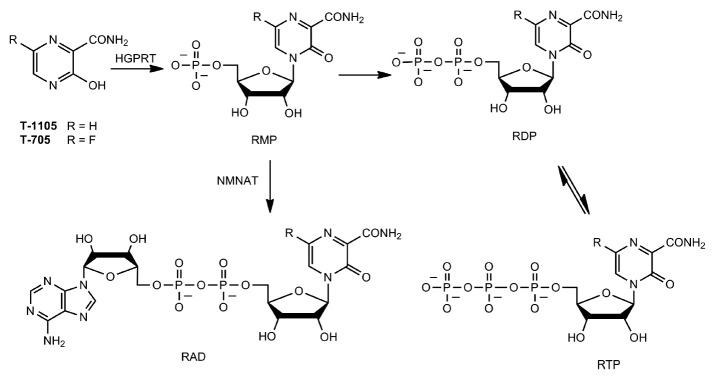
Scheme for the conversion of T-705 and T-1105 to their active metabolites, ribose-5’-triphosphates (RTPs), and a parallel pathway in which monophosphate forms (RMPs) of T-705 and T-1105 are converted to the RAD metabolite

Similar to favipiravir, T-1105, as the active nucleoside 5’-triphosphate (T-1105-RTP) form, inhibits viral RdRp. Compared with favipiravir, the efficiency of* in vitro *activation of 3-hydroxypyrazine-2-carboxamide to its ribose-5’-triphosphate form is more dependent on the cell line in which this activation occurs. For example, T-1105 showed higher antiviral activity in MDCK cells (the T-1105-RTP level was 841 and 1,228 pmol/106 cells after 24-hour incubation with 0.5 and 1 mM T-1105, respectively). In the control experiment in this cell line at equimolar favipiravir concentrations of 0.5 and 1 mM, the T-705-RTP level was 4-fold lower than the T-1105-RTP level. Antiviral activity of T-1105-RTP was not detected in A549 and Vero cells (less than 50 pmol/106 cells), as well as in HEK293T cells (65 and 171 pmol/106 cells after 24- hour incubation with 0.5 and 1 mM T-1105, respectively). In these three cell lines, T-1105 activation was hampered by an inefficient conversion of T-1105-RMP to T-1105-RDP. This phenomenon is associated with the fact that the main metabolic pathway is accompanied by a parallel reaction converting T-1105-RMP to the T-1105-RAD metabolite with nicotinamide mononucleotide adenylyltransferase (NMNAT) (*Fig. 4*) [56].

Because T-705-RAD and T-1105-RAD are found in all of the described cell lines, they are being studied as nicotinamide adenine dinucleotide (NAD) analogs.

The non-fluorinated analog of favipiravir, T-1105, was found to be active against the Chikungunya virus (CHIKV) *in vitro*. In that case, T-1105 was a selective inhibitor of the cytopathogenic effect induced by clinical isolates of CHIKV and other alphaviruses. The antiviral activity of T-1105 was 2- to 5-fold higher than that of favipiravir. For example, for the CHIKV Indian Ocean strain 899 (lab), the EC_50_ value was 25 ± 3 µmol/L for T-705 and 7.0 ± 1 µmol/L for T-1105 [27].

In *in vivo *experiments with the foot-and-mouth disease virus, T-1105 efficiently suppressed the clinical signs of the disease in infected pigs and reduced viremia and virus shedding (oral dose: 400 mg/kg/day for 6 days). The efficacy of 3-hydroxypyrazine-2-carboxamide and the prophylactic O1 Manisa vaccine against the foot-and-mouth disease virus was also compared in a guinea pig model. The efficacy of prophylactic therapy with T-1105 (guinea pigs, 400 mg/kg/day orally for 5 days) was shown to be comparable to that of animal vaccination [57].

T-1106 proved more efficient than favipiravir against the yellow fever virus (YFV) in a Syrian hamster model with a minimum effective dose of 32 mg/kg/day administered intraperitoneally or orally. T-1106 had no antiviral activity in experiments on the cytopathic effect induced by the yellow fever virus in the Vero (EC_50_ more than 100 µg/mL) and CV-1 (EC_50_ more than 369 µmol/L) cell lines [58, 59].

Favipiravir was more efficient than T-1106 *in vitro* against several members of the Phlebovirus genus. At the same time, the efficacy of T-1106 in a model of Syrian hamsters infected with the Punta Toro virus, which is characterized by liver damage, was 9.4-fold higher than that of favipiravir (based on ED50). In a mouse model, favipiravir showed the best antiviral activity [60].

The activity of the T-1105 and T-1106 nucleosides against the dengue virus (DENV) was compared* in vitro *[32]. The efficacy of T-1105 (EC_50_ 21 ± 0.7 µmol/L) was 5-fold higher than the EC_50_ of favipiravir. It exceeded the activity of T-1106 by almost the same factor (EC_50_ 113 ± 11 µmol/L for T-1106). In addition, both T-1105 and its nucleoside are capable of inducing lethal mutagenesis of the viral genome due to base mispairing during the formation of the RNA secondary structure.

Obviously, high activity against RNA viruses is inherent not only to favipiravir, but also to its structural analogs. A number of studies have shown an even higher efficacy of T-1105 and T-1106 compared to that of favipiravir both *in vitro *and *in vivo*, which points to the need for their clinical study for further use as antiviral drugs.

## SYNTHESIS OF FAVIPIRAVIR AND ITS DERIVATIVES

Classical synthetic approaches to the production of favipiravir are described in detail in three recent reviews by Y. Titova [61], N. Al Bujug [62], and W. Hu [63].

**Fig. 5:**
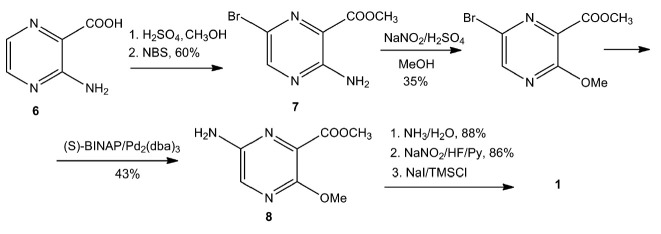
Synthesis of favipiravir (**1**) according to the strategy developed by Y. Furuta (Toyama Company)

The first version of favipiravir synthesis was patented and then published by Y. Furuta et al. from Toyama Chemical Company (*Fig. 5*) [64]. The starting 3-aminopyrazine-2-carboxylic acid (**6**) was first esterified and then brominated to yield aminocarboxylate (**7**). The formation of aminopyrazine (**8**) using an expensive Pd2/diphenylphosphino-binaphthyl (BINAP) catalyst occurred with a low yield of 43%. The second bottleneck of this technology was the use of Olah’s reagent (HF/Py) to introduce the F atom into position 6 of the base. The overall yield of favipiravir did not exceed 1%. This technology is very difficult to scaleup.

**Fig. 6:**
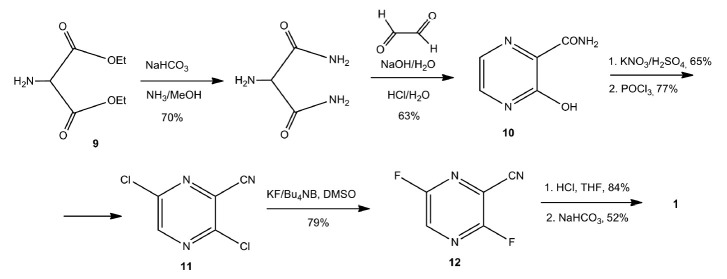
Improved synthesis of favipiravir (**1**) following the Y. Furuta strategy (Toyama Company)

Another route of favipiravir synthesis was proposed by the same authors in 2001 (*Fig. 6*) [65].

The starting compound in that synthesis was the available aminomalonic acid diethyl ester (**9**) that was converted into 3-hydroxypyrazine-2-carboxamide (**10**) in two steps. The latter was converted into favipiravir (**1**) in a series of consecutive transformations of functional groups. The overall yield of the product was 17%.

**Fig. 7:**
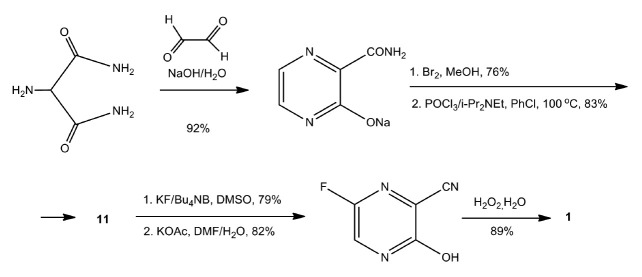
Modified version of the synthesis of favipiravir (**1**) following the Nippon Soda & Toyama Company strategy (2011)

A modified version of the latter synthesis of favipiravir was developed by Toyama in collaboration with Nippon Soda Corporation [66, 67] (*Fig. 7*). Using this method, it was possible to synthesize favipiravir with an overall yield of 33%.

**Fig. 8:**
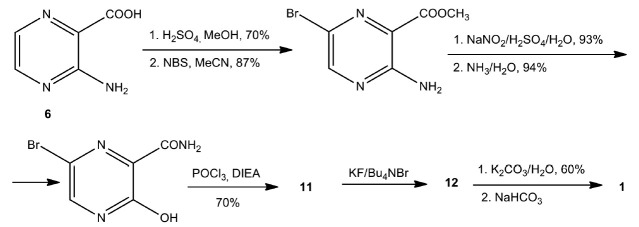
Synthesis of favipiravir (**1**) according to the Liu Feng procedure

The fourth version of favipiravir synthesis was proposed by Liu Feng et al. in 2017 [68] (*Fig. 8*). All intermediate products were purified by crystallization; the last step was performed in one pot; favipiravir (**1**) was easily isolated by recrystallization. However, this synthesis uses a large amount of phosphorus oxychloride, which constitutes a problem for the scaling up of the process, acting as an environmental pollution factor.

In addition, 3,6-dichloropyrazine-2-nitrile (**11**) is a strong allergen that causes skin irritation. Because of these factors, Liu Feng’s technology was not scaled up to industrial production of favipiravir.

**Fig. 9:**
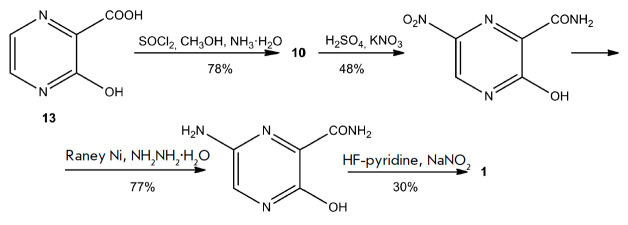
Synthesis of 6-fluoro-3-hydroxypyrazine- 2-carboxamide (**1**) according to the Zhang strategy

Favipiravir can be produced by the four-step method, proposed by Zhang et al., from commercially available 3-hydroxypyrazine-2-carboxylic acid (**13**) through amidation, nitration, reduction, and fluorination (*Fig. 9*) [69].

**Fig. 10:**
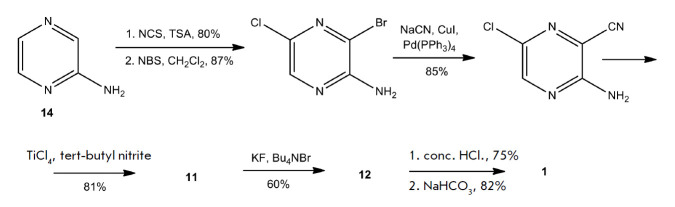
Synthesis of favipiravir according to the Xie method (2019). NCS – N-chlorosuccinimide, TSA – *p*-toluenesulfonamide

Another approach to favipiravir synthesis was proposed by Xie et al. [70]. This approach was to produce T-705 from inexpensive and widely available 2-aminopyrazine (**14**). A four-step synthesis of an intermediate compound, 3,6-dichloropyrazine-2-carbonitrile (**11**), was developed, which did not require the use of POCl3 and afforded a good yield of the product (*Fig. 10*).

**Fig. 11:**
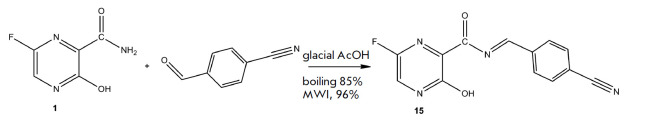
Synthesis of the 4-cyanobenzylidene analog (**15**) of favipiravir. MWI – microwave irradiation

In 2021, synthesis of (E)-N-(4-cyanobenzylidene)- 6-fluoro-3-hydroxypyrazine-2-carboxamide ((**15**), Cyanorona-20) was reported [71] (*Fig. 11*). The authors claimed it was the first selective SARS-CoV-2 RdRp inhibitor 209-fold more efficient than favipiravir* in vitro *(EC_50_ = 0.45 μM, EC_50_ (T-705) = 94.09 μM).

Pre-synthesis computational studies predicted that compound (**15**) may act as an inhibitor of SARSCoV- 2 RdRp through the formation of riboside-5’-triphosphate via the mechanism described for favipiraPre-synthesis computational studies predicted that compound (**15**) may act as an inhibitor of SARSCoV- 2 RdRp through the formation of riboside-5’-triphosphate via the mechanism described for favipiravir. In addition, the cyano group is a zincophore; i.e., it can be a carrier of zinc ions, reducing its intracellular concentration. Zn^2+^ is a SARS-CoV-2 RdRp cofactor, and a decrease in its concentration drastically affects RdRp activity. The lipophilic benzylidene moiety of Cyanorona-20 promotes better transfer through the cytoplasmic membrane of the cell. However, the paper failed to report data on any changes in the solubility of the base (**15**) compared to that of T-705; it only stated that the results of Cyanorona-20 aqueous dissolution testing were excellent [71].

**Fig. 12:**
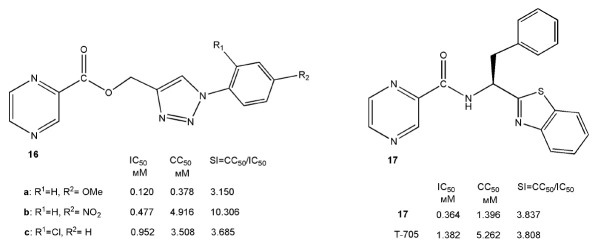
Pyrazine-triazole (**16**) and pyrazinebenzothiazole (**17**) analogs of favipiravir, which exhibit antiviral activity against SARS-CoV-2

There were attempts to synthesize new analogs of 2-pyrazinecarboxamide to enhance antiviral activity against SARS-CoV-2 [72]. A series of seven pyrazine- triazole (**16**) and 11 pyrazine-benzothiazole (**17**) heterocyclic bases was synthesized. *Figure 12* shows analogs of favipiravir (**1**) with comparable or better antiviral properties.

**Fig. 13:**
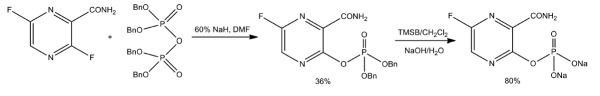
Synthesis of favipiravir phosphate

An attempt was made to improve the solubility and bioavailability of favipiravir by a synthesis of its phosphate (*Fig. 13*) [73]. However, no data was offered on the antiviral activity of the produced compounds.

**Fig. 14:**
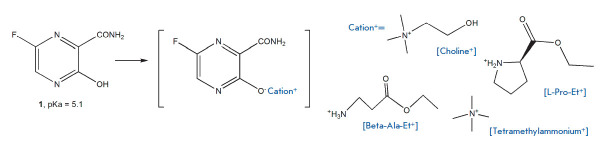
Ionic liquid-based formulation of favipiravir (**1**)

Another approach to improving the solubility of favipiravir was used by a group of Japanese researchers [74]. They tried to solubilize poorly soluble favipiravir using counterions of ethyl esters of L-proline (L-Pro-Et+) and beta-alanine (Beta-Ala-Et+), choline chloride, and tetramethylammonium hydrochloride (*Fig. 14*). The method is now used in pharma to produce poorly soluble active pharmaceutical substances or proteins balanced with various counterions [75]. According to NMR, the stoichiometric ratio of T-705 and the counterions was 1 : 1. The produced ionic liquid-based formulation of favipiravir (like in the original article) were amorphous (according to X-ray diffraction analysis) and had significantly better water solubility compared with that of the original crystalline favipiravir: the choline counterion was characterized by the best solubility (739 mg/mL for Cho-T-705 versus 7.0 mg/mL for T-705, *Fig. 14*).

In formulations *in vivo *experiments, all ionic liquid- based formulation of favipiravir had better pharmacokinetic and pharmacodynamic characteristics compared with those of the original favipiravir [74].

**Fig. 15:**
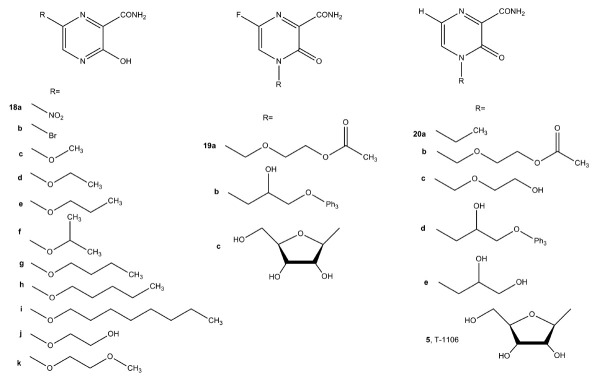
Favipiravir analogs for studying activity against the Zika virus

There have been attempts to synthesize effective drugs based on 3-oxopyrazine-2-carboxamide against the Zika virus (*Fig. 15*) [76]. 3-Hydroxypyrazine-2- carboxamide and favipiravir displayed antiviral activity against the Zika virus in the Vero cell line. T-1105 significantly reduced the level of cell death (EC_50_ = 97.5 ± 6.8 µmol/L).

Testing of analogs (**18**)–(**20**) showed very low (CC50 = 200–300 µmol/L for compounds (**18f–i**)) or no (CC50 >1000 µmol/L) antiviral activity [76].

**Fig. 16:**
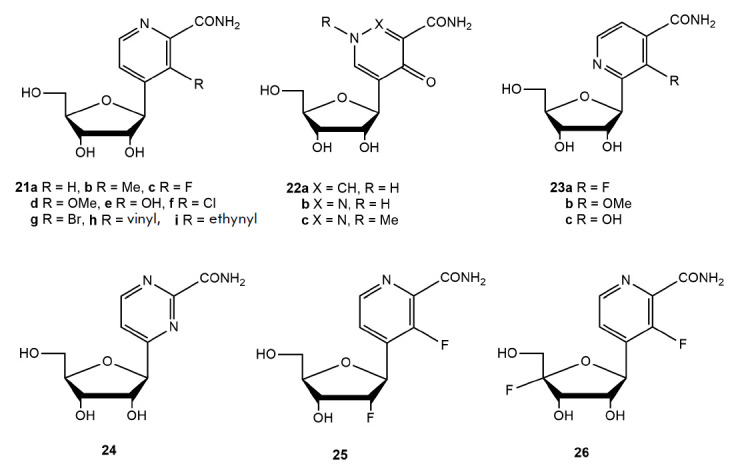
C-nucleoside derivatives of pyridine, pyridazine, and pyrimidine

Wang et al. [77] synthesized a series of pyridine, pyridazine, and pyrimidine C-nucleosides, analogs of favipiravir (*Fig. 16*).

The antiviral activity of all the compounds was studied in MDCK cells infected with the influenza virus A/WSN/33 (H1N1). Compound (**21e**) exhibited the highest activity (EC_50_ = 1.3 µmol/L). At the same time, this compound had high cytotoxicity: the 50% cytotoxic concentration (CC50) was 2.0 µmol/L. The antiviral activity of compound (**21c**) was comparable to that of T-705: the EC_50_ was 1.9 µmol/L, and the CC50 was more than 400 µmol/L. The remaining C-nucleosides showed low or weak antiviral activity; even compounds (**25**) and (**26**) with a modification at the positions of the 2’-OH and 4’-H-group of ribose had low activity [77].

**Fig. 17:**
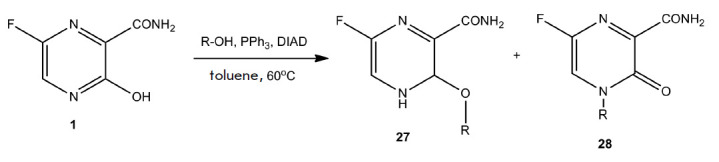
General scheme of favipiravir alkylation under Mitsunobu reaction conditions. R-OH –hydroxyalkyl phosphonates

The synthesis of acyclic nucleotide analogs of favipiravir as potential inhibitors of hypoxanthine-guanine- xanthine phosphoribosyltransferase (HGXPRT) from malarial *Plasmodium falciparum *was proposed [78]. HGXPRT catalyzes the magnesium-dependent synthesis of nucleoside 5’-monophosphates from purine bases (guanine or hypoxanthine). The acyclic nucleotide analogs (**27**) and (**28**) were synthesized from favipiravir via the Mitsunobu reaction. Alkylation occurred at positions N4 or O3 of the heterocyclic ring to form the N-(**28**) or O-regioisomer (**27**) (*Fig. 17*).

**Fig. 18:**
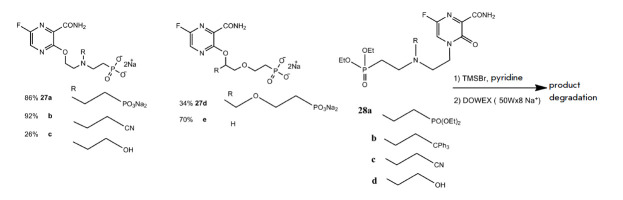
Acyclic nucleotide derivatives of favipiravir

O-alkylated acyclic nucleotide derivatives of favipiravir (**27**) were produced according to the scheme shown in *Fig. 18*. Unfortunately, the N-alkylated derivatives of T-705 were unstable under deprotection conditions.

Investigation of the O-alkylated acyclic nucleotide derivatives of favipiravir as inhibitors of human HGPRT and *Pf*HGXPRT showed that none of the compounds inhibited any enzyme in the concentration range of 100 to 150 µmol/L. Acyclic nucleotide derivatives of guanine or hypoxanthine with the same substituents are efficient inhibitors of the HGPRT and* Pf*HGXPRT enzymes, with the inhibition constant ranging from 0.07 to 5 µmol/L [78].

**Fig. 19:**
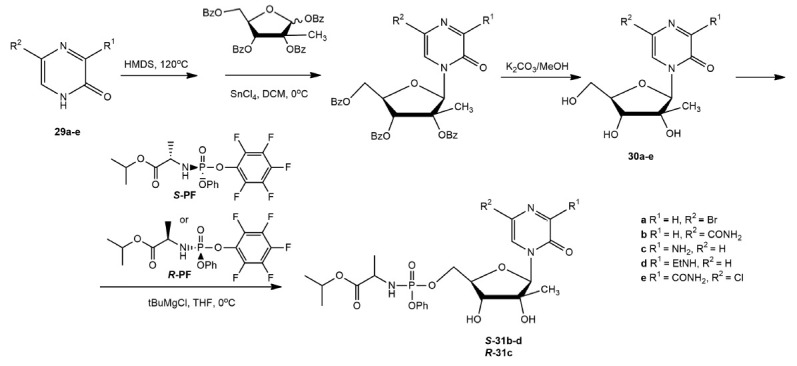
Synthesis of 3-oxo-4-(2-C-methyl-β-*D*-ribofuranosyl)-pyrazines and their 5’-phosphoramidate prodrugs

Synthesis of nucleoside-based prodrugs is a modern approach to the production of new antiviral drugs [79]. Synthesis of several pyrazine nucleosides and their phosphoramidate prodrugs was described in (*Fig. 19*) [80]. The activity of these nucleosides against the hepatitis C virus (HCV) was evaluated. 3-oxo-4-(2-C-methyl-β-*D*-ribofuranosyl)-pyrazines and their 5’-phosphoramidate prodrugs were synthesized using the silyl method by glycosylation of the bases (**29a–e**) with 1,2,3,5-tetra-O-benzoyl-2-Cmethyl- β-D-ribofuranose in the presence of tin tetrachloride (SnCl4). After removal of benzoyl (Bz) protecting groups, phosphoramidate derivatives were synthesized by reacting with *S*-PF or the (*R*)-2-((*R*)- (2,3,4,5,6-pentafluorophenoxy)phenoxyphosphorylamino) propionic acid isopropyl ester (*R*-PF).

**Fig. 20:**
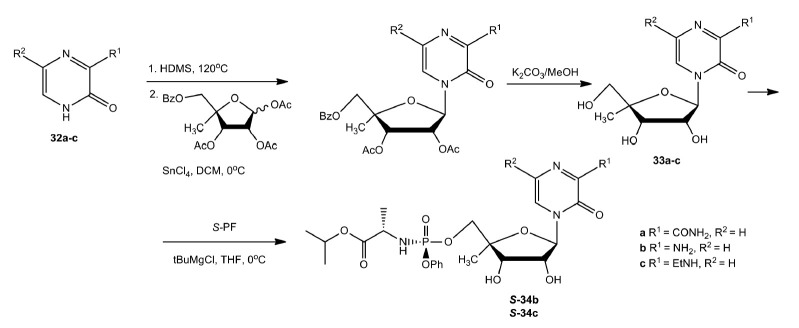
Synthesis of 3-oxo-4-(4-C-methyl-β-*D*-ribofuranosyl)-pyrazines and their 5’-phosphoramidate prodrugs

3-Oxo-4-(4-C-methyl-β-D-ribofuranosyl)-pyrazines and their 5’-phosphoramidate prodrugs were similarly synthesized from the corresponding bases (*Fig. 20*).

*In vitro *investigation of the activity of synthesized compounds against HCV showed that among the compounds (**30a–d**), only (**30c**) demonstrated a low inhibition rate of 22.3% at a concentration of 100 µmol/L. The ethylamine group at position C3 of the heterocyclic ring caused a loss of the antiviral activity of compound (**30d**) and its (*S*)-phosphoramidate (**S-31d**). Compound (**30e**) showed good activity with an EC_50_ value of 7.3 µmol/L; however, attempts to convert it to a phosphoramidate prodrug failed [80].

It was presumed that changing the position of the methyl group in the ribose moiety (compounds (**33a–c**)) may reduce their cytotoxicity. However, among these compounds, only the (*S*)-isomer phosphoramidate prodrug (**S-34b**) was not cytotoxic at a concentration of 100 µmol/L, but it showed weak activity (EC_50_ = 19.5 µM) [80].

**Fig. 21:**
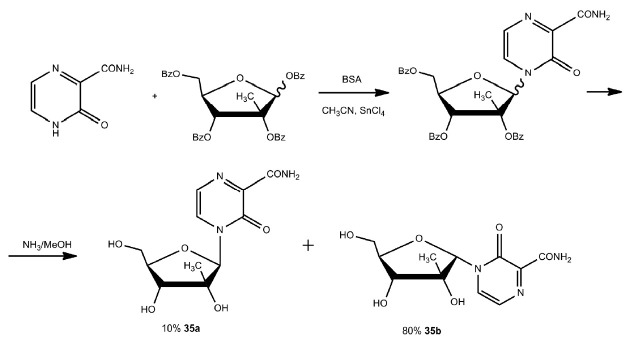
Synthesis of 3-oxo-4-(β-*D*ribofuranosyl)- 2- pyrazinecarboxamide. BSA – N,Obis( trimethylsilyl) acetamide

The nonfluorinated base T-1105 was used to synthesize 3-oxo-4-(2-C-methyl-β-D-ribofuranosyl)-2- pyrazinecarboxamide (*Fig. 21*) as an α/β-anomeric mixture. After ammonolysis of the benzoyl (Bz) protecting groups, the desired β-anomeric product (**35a**) was isolated at a yield of 10% only and the α-anomer (**35b**) was also isolated at a yield of 58% [81].

Unfortunately, the nucleosides (**35a**) and (**35b**) showed neither antiviral activity against RNA viruses nor cytotoxicity *in vitro *at concentrations up to 100 µmol/L [81].

**Fig. 22:**
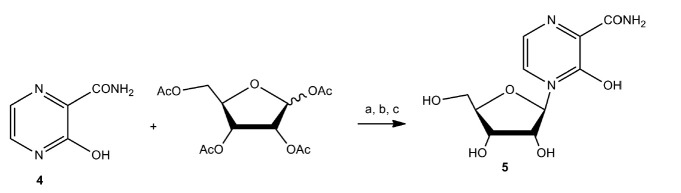
Synthesis of 3-oxo-4-(β-*D*-ribofuranosyl)-2-pyrazinecarboxamide (**5**). a) 1,2,3,5-tetra-O-acetyl-β-*D*ribofuranose, N,O-bis(trimethylsilyl)acetamide, acetonitrile, 30 min, rt; b) trimethylsilyl trifluoromethanesulfonate, acetonitrile, 44 h, rt; c) methanol, water, triethylamine, 6 h, rt

Typically, classical chemical glycosylation methods are used in the synthesis of modified nucleosides and nucleotides based on T-705 and T-1105. For example, 3-oxo-4-(β-*D*-ribofuranosyl)-2-pyrazinecarboxamide (**5**) (*Fig. 22*) is synthesized following the Vorbruggen procedure by treating 3-hydroxypyrazine-2-carboxamide (T-1105) with 1,2,3,5-tetra-O-acetyl-β-*D*ribofuranose in anhydrous acetonitrile (CH_3_CN) in the presence of N,O-bis(trimethylsilyl)acetamide at room temperature, followed by the addition of trimethylsilyl trifluoromethanesulfonate. The yield of the desired product in this procedure is 55% [82].

**Fig. 23:**
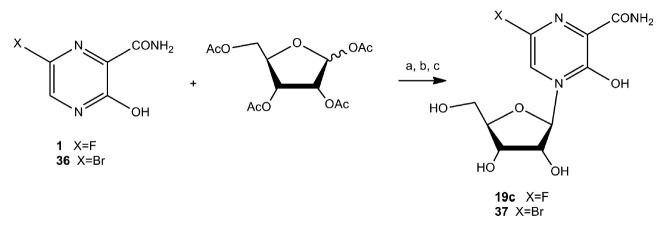
Synthesis of

Chemical synthesis of 6-fluoro-3-oxo-4-(β-Dribofuranosyl)-2-pyrazine-carboxamide (**19c**) (*Fig. 23*) can be performed by treating C6-substituted 3-hydroxypyrazine- 2-carboxamide with ammonium sulfate (NH_4_)_2_SO_4_ in hexamethyldisilazane at 140°C.

The resulting silylated pyrazinecarboxamide is reacted with peracylated ribofuranose in the presence of tin tetrachloride (SnCl4). The yield of pure nucleoside after chromatographic purification is 40%. Transfer of optimized conditions for the synthesis of 6-fluoro-3-oxo-4-(β-D-ribofuranosyl)-2- pyrazinecarboxamide to the 6-bromo-substituted analog of favipiravir provided compound (**37**) in a yield of 68% [82].

**Fig. 24:**
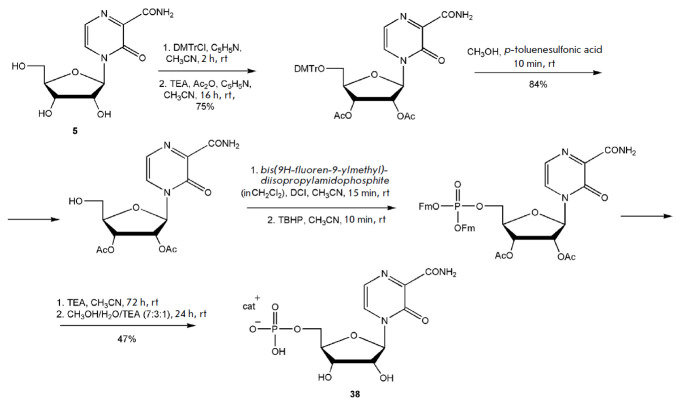
Synthesis of T-1105 riboside phosphate. DCI – dicyanoimidazole; TEA – triethylamine

In 2018, J. Huchting presented a scheme for the synthesis of T-1105 riboside phosphate (**38**) (*Fig. 24*) and a similar method for the synthesis of the favipiravir nucleotide [83]. Huchting et al. were able to chemically synthesize nucleoside 5’-monophosphate, diphosphate, and triphosphate of T-1105.

The most efficient route for the synthesis of 3-oxo-4-(β-*D*-ribofuranosyl-5’-phosphate)-2- pyrazinecarboxamide (**33**) was by phosphorylation of compound (**5**) with preliminary protection of the 2’- and 3’-OH groups of ribose. The yield in the target compound (**38**) was 47% [83].

**Fig. 25:**
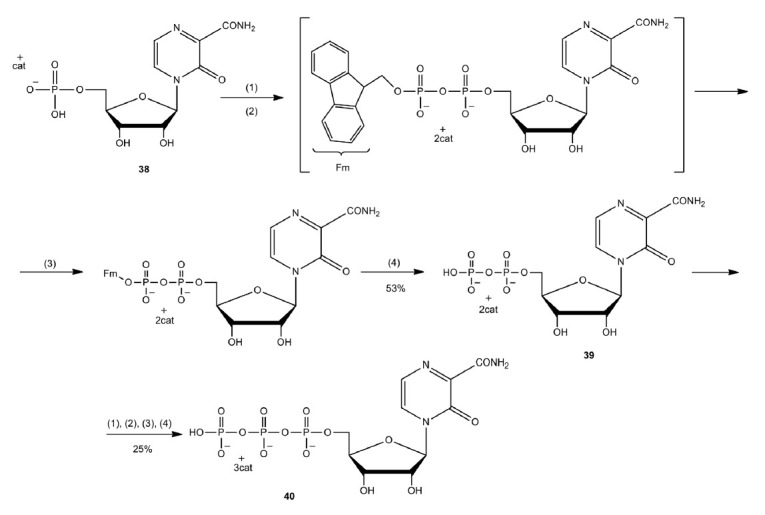
Synthesis of

Nucleoside 5’-diphosphate and triphosphate of T-1105 were prepared by sequential two-step synthesis using fluorenylmethyl (Fm) protecting groups (*Fig. 25*).

**Fig. 26:**
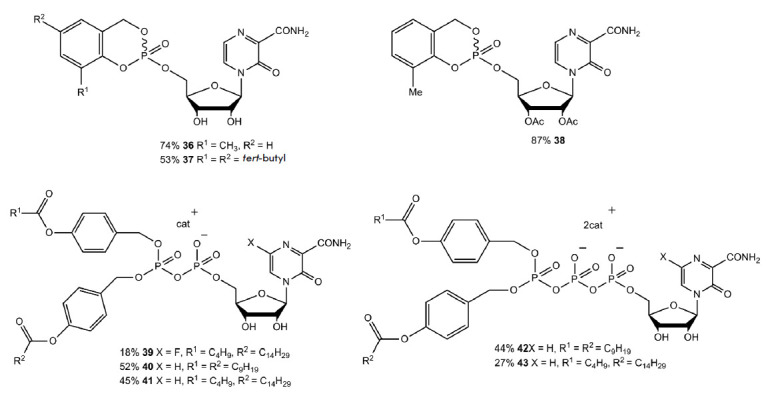
Structural formulas of c*yclo*Sal pronucleotides (**28**)–(**30**) and Di*PP*ro and Tri*PPP*ro of compounds (**31**)–(**35**)

To improve lipophilicity and screen negatively charged groups of the T-1105 and T-705 nucleotides, depot forms of *cyclo*Sal-pronucleotides, Di*PP*ro, and Tri*PPP*ro were synthesized (*Fig. 26*). *Cyclo*Salpronucleotides are prodrugs; controlled release of active nucleotides occurs in a pH-dependent manner. They were produced using phosphoramidite synthesis. Activation of the Di*PP*ro and Tri*PPP*ro prodrugs includes a major and minor pathway. The major pathway is activation of these compounds by esterases and subsequent efficient release of nucleotides 83].

The minor metabolic pathway involves hydrolytic cleavage of phosphoanhydride in the pronucleotide, which leads to the formation of an undesired nucleotide [83]. The antiviral activity of these compounds was tested in MDCK and MDCK-TGres (HGPRTdeficient cell line) cells using two influenza strains: A/X-31 (A/H3N2 subtype) and B/Ned/537/05. The cytotoxicity of these compounds was evaluated in uninfected cells. Compound (**36**) exhibited the highest antiviral activity and minimal toxicity. The mean EC_50_ was 0.91 µmol/L in MDCK cells. All Di*PP*ro and Tri*PPP*ro compounds retained antiviral activity in MDCK-TGres cells. For example, the mean EC_50_ value for compound (**36**) in MDCK-TGres cells was 0.80 µmol/L [83].

Obviously, the development of simple and efficient enzymatic methods for the synthesis of modified nucleosides and nucleotides based on 3-hydroxypyrazine- 2-carboxamide and its 6-fluoro-substituted analog is extremely important.

**Fig. 27:**
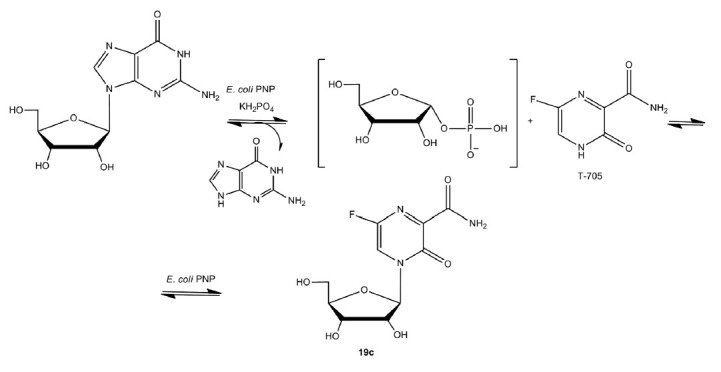
Enzymatic synthesis of T-705 riboside

To date, there exists only one short communication on the enzymatic synthesis of modified nucleosides based on substituted 3-hydroxypyrazine-2-carboxamides using *E. coli *purine nucleoside phosphorylase (PNP) [84]. The efficiency in the transfer of the T-705 base to the ribose moiety reached 43% in 4 h (*Fig. 27*). However, the study did not provide the yield and spectral characteristics of the product.

Ribosyltransferases are involved in the formation of all C-N-glycosidic bonds in nucleoside monophosphates via the *de novo *biosynthetic pathway. Purine phosphoribosyltransferases catalyze a reversible transfer of the 5-phosphoribosyl group from PRPP to nitrogen at position 9 in 6-amino- or 6-oxopurines in the presence of Mg^2+^ to form the corresponding ribose-5’-monophosphate [85].

According to substrate specificity, there are 6-aminopurine and 6-oxopurine purine phosphoribosyltransferases, (APRTs) and (HPRTs, HGPRTs, etc.), respectively. APRTs are strictly specific to 6-aminopurines, such as adenine, 2-fluoroadenine, or 2-chloroadenine. 6-oxopurine PRTs can recognize various 6-oxopurines, such as hypoxanthine, guanine, xanthine, and other 6-oxo- and 6-mercaptopurine analogs [85].

**Fig. 28:**
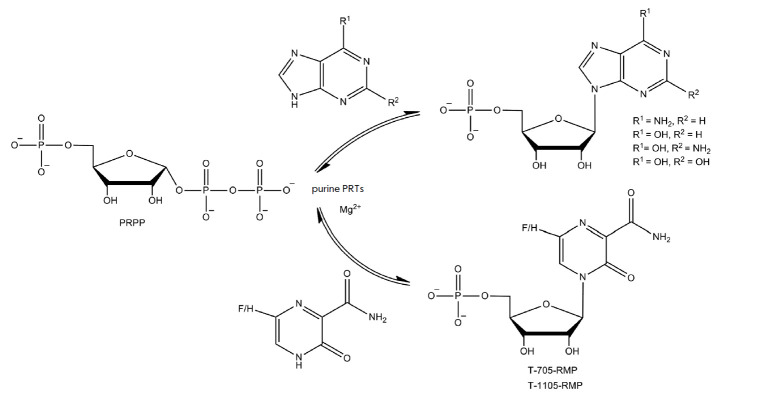
Enzymatic synthesis of ribose-5’-monophosphates catalyzed by phosphoribosyl transferases (PRTs)

The active metabolite of favipiravir and 3-hydroxypyrazine- 2-carboxamide is their ribose-5’-triphosphate form that is involved in the suppression of the activity of RNA viruses. Naesens et al. [21] found that in the cell, human HGPRT first phosphorylates T-705 into 6-fluoro-3-oxo-4-(β-*D*-ribofuranosyl-5’- phosphate)-2-pyrazinecarboxamide (T-705-RMP) and T-1105 into 3-oxo-4-(β-*D*-ribofuranosyl-5’-phosphate)- 2-pyrazinecarboxamide (T-1105-RMP) (*Fig. 28*). However, T-705 and T-1105 show low affinity for the HGPRT active site under both synthesis and intracellular phosphoribosylation conditions. Human APRT was found to catalyze T-705 and T-1105 phosphoribosylation 40-fold less efficiently than HGPRT under similar conditions. In addition, these researchers found that T-705 and T-1105 were poor substrates for human PNP [21].

An extract of MDCK cells is known to be used to assess the metabolic activation profiles of favipiravir and 3-hydroxypyrazine-2-carboxamide [83]. Phosphoribosylation of favipiravir in a MDCK cell extract is less efficient than that of 3-hydroxypyrazine- 2-carboxamide. The formation of the T-705- RMP metabolite in the MDCK cell extract upon incubation of T-705 with 5-phosphoribosyl-α-1- pyrophosphate (PRPP) was 35% after 25 h of incubation. The yield of the T-1105-RMP metabolite in the MDCK cell extract during the incubation of T-1105 with PRPP was 90% after 19 h of incubation. Further incubation of T-1105-RMP with the MDCK cell extract for 15 h did not result in the formation of either T-1105-RDP or T-1105-RTP, even with the addition of a high concentration of ATP (phosphate donor). However, incubation of T-1105-RDP with a 10-fold higher ATP concentration led to its effective phosphorylation: T-1105-RTP formed 2 min after incubation and remained the main metabolite for the next 2 h.

Single examples of the biosynthesis of pyrazinecarboxamide nucleosides and nucleotides indicate that classical chemical methods remain the main routes for their synthesis.

## CONCLUSION

Favipiravir T-705 and some of its structural analogs exert a significant antiviral effect against RNA viruses. However, a high dose load (up to 3.6 g of favipiravir per day in the treatment of COVID-19), poor bioavailability due to low solubility, high systemic toxicity, and the teratogenic activity of the drug encourage researchers to continue synthesizing more and more new structural analogs in an effort to increase the selectivity of the active molecule and reduce its toxicity.

Most likely, the use of the nucleosides of favipiravir and its structural analogs may reduce the dose load on the human body and reduce the toxic effect of the drug. To date, many pyrazinecarboxamide nucleosides modified in the heterocyclic base and carbohydrate moiety have been synthesized. A series of acyclic linear analogs and nucleosides modified in the ribose 5’-hydroxyl group has been produced. However, the efficacy of these compounds in the treatment of human viral infections has yet to be proven.

Therefore, the structural analogs of 1,4-pyrazine- 3-carboxamide may become the basis for the development of new selective and highly effective antiviral drugs to be used during viral pandemics and, in some cases, extremely severe viral infections.

## Abbreviations

APRT: adenine phosphoribosyltransferase
Bz: benzoyl group
C: cytidine
CHIKV: Chikungunya virus
CC50: 50% cytotoxic concentration
CT: computed tomography
DCI: 4,5-dicyanoimidazole
DENV: dengue virus
EBOV: Ebola virus
EC50: 50% effective concentration
GTP: guanosine 5’-triphosphate
G: guanosine
HCV: hepatitis C virus
HEK-293 cells: human embryonic kidney 293 cells
HGPRT: hypoxanthine-guanine phosphoribosyltransferase
HPRT: hypoxanthine phosphoribosyltransferase
HGXPRT: hypoxanthine guanine xanthine phosphoribosyltransferase
MDCK cells: Madin-Darby canine kidney cells
MV: mechanical ventilation
NAD: nicotinamide adenine dinucleotide
NCS: N-chlorosuccinimide
NMNAT: nicotinamide mononucleotide adenylyltransferase
PFU: plaque forming unit
PNP: purine nucleoside phosphorylase
PRPP: 5-phospho-ribosyl-1-alpha-pyrophosphate
RdRp: RNA-dependent RNA polymerase
RDP: ribose-5’-diphosphate
RMP: ribose 5’-monophosphate
RTP: ribose 5’-triphosphate
SARS: severe acute respiratory syndrome
SI: selectivity index (CC50/EC50)
SOC: standard of care
T-1105: 3-hydroxypyrazine-2-carboxamide
T-1106: 3-oxo-4-(β-D-ribofuranosyl)- 2-pyrazinecarboxamide
T-705: 6-fluoro-3-hydroxypyrazine-2-carboxamide
TCID50: 50% tissue culture infectious dose
TSA: p-toluenesulfonamide
YFV: yellow fever virus.
